# Assessment of the Therapeutic Potential of Hesperidin and Quercetin Nanoparticles on Oxidative Stress, Enzyme Activities, Trophic Factors, and Monoamines in an Animal Model of Depression

**DOI:** 10.1007/s11064-025-04447-2

**Published:** 2025-06-16

**Authors:** Haitham S. Mohammed, Eman N. Hosny, Hussein G Sawie, Yasser A. Khadrawy

**Affiliations:** 1 Biophysics Department, Faculty of Science, Cairo University, Giza, Egypt; 2 Medical Physiology Department, National research Centre, Giza, Egypt

**Keywords:** Depression, Oxidative stress, BDNF, Monoamines, Hesperidin, Quercetin

## Abstract

Natural remedies have emerged as promising alternative or complementary therapies for combating depression. This study aimed to investigate the effects of hesperidin (HSP-NPs) and quercetin (QUR-NPs) nanoparticles on oxidative stress markers, enzyme activities, brain-derived neurotrophic factor, and monoamine levels in the cortex and hippocampus of a reserpine-induced rat model of depression. The depression model was established by administering reserpine (0.2 mg/kg) to the animals for 25 days. On the 26^th,^ rats were administered i.p. with reserpine (0.1 mg/kg) and oral isotonic saline solution for another 21 days. Following reserpine administration, the animals exhibited a significant reduction in nitric oxide (NO), reduced glutathione (GSH), brain-derived neurotrophic factor (BDNF), serotonin (5-HT), norepinephrine (NE), and dopamine (DA) levels. Additionally, a significant decrease in Na, K,ATPase activity and a significant increase in acetylcholinesterase (AchE) and monoamine oxidase (MAO) activity were observed in the cortex and hippocampus as compared to the control group of animals. Treatment with either HSP-NPs and QUR-NPs for 14 days mitigated the oxidative stress in the cortex and hippocampus. The treatments restored the changes Na, K,ATPase, and MAO, AchE activities in the two brain regions. Notably, QUR-NPs was superior in alleviating the adverse changes induced by reserpine concerning monoamines and BDNF levels. These findings highlight the therapeutic potential of HSP-NPs and QUR-NPs in mitigating the underlying etiology of depressive symptoms rat model. Further investigations are warranted to exploAuthorre the potential synergistic effects of hesperidin and quercetin when used in combination at different doses and treatment durations.

## Introduction

Depression affects millions worldwide, causing significant personal suffering and societal burden [1]. While various pharmacological agents are available for the treatment of depression, their efficacy and tolerability are still areas of concern. Moreover, the long-term use of synthetic antidepressant drugs may be associated with side effects and limited response rates in some individuals [2]. Natural materials derived from plants, such as flavonoids, have gained attention due to their diverse biological activities, including antioxidant, anti-inflammatory, and neuroprotective effects [3]. Among these flavonoids, hesperidin, and quercetin have shown promise in preclinical and clinical studies for their potential antidepressant effects [4, 5].

Hesperidin, primarily found in citrus fruits, possesses antioxidant and anti-inflammatory properties and has demonstrated antidepressant-like effects in animal models of depression [3]. Quercetin, abundant in various fruits and vegetables, exhibits antioxidant and anti-inflammatory actions and has shown antidepressant-like effects in preclinical studies [6]. However, using hesperidin and quercetin in native form put several constraints on their efficacy particularly in in-vivo applications [7, 8]. The nanoparticle formulation of these compounds offers several advantages in their in-vivo application including enhanced dissolution and solubility, improved bioavailability, dose reduction, and higher therapeutic efficacy [9].

Studies have shown that individuals with depression often exhibit increased markers of oxidative stress in the cortex and hippocampus, including elevated levels of lipid peroxidation and decreased antioxidant enzyme activities [10]. This oxidative imbalance can lead to cellular damage and impair neuronal function, contributing to the pathophysiology of depression [11]. Monoamines, such as serotonin, norepinephrine, and dopamine, are neurotransmitters involved in regulating mood, emotions, and cognition [12]. In depression, there is evidence of altered monoamine levels and dysregulation of their signaling pathways in the cortex and hippocampus. Reduced levels of serotonin, for example, have been associated with depressive symptoms. These alterations in monoamine neurotransmitters can disrupt neural circuits involved in mood regulation and contribute to the development and maintenance of depression [13]. Neuroenzymes, including monoamine oxidase (MAO) and acetylcholinesterase (AChE), play crucial roles in the metabolism and breakdown of neurotransmitters in the brain. Studies have reported changes in the activities of these neuroenzymes in the cortex and hippocampus of individuals with depression. Increased MAO activity, for instance, can result in the breakdown of monoamines, further exacerbating the monoamine imbalance observed in depression [14]. Similarly, altered AChE activity may impact cholinergic neurotransmission, which is implicated in mood regulation and cognitive processes [15]. Na, K-ATPase is an enzyme found in the cell membrane of neurons and plays a crucial role in maintaining ion homeostasis [16]. The activity of this enzyme affects the balance between sodium and potassium ions, neuronal excitability, and neurotransmitter regulation [17]. Therefore, alteration in the activity of this enzyme may potentially contribute to depressive symptoms.

Reserpine, a drug that depletes serotonin, norepinephrine, and dopamine [18, 19] is commonly used to induce depressive-like behaviors in animal models [20]. In light of the existing evidence, this study aims to investigate the effects of HSP-NPs and QUR-NPs on depression symptoms using an animal model.

## Materials and methods

### Chemicals

Hesperidin, quercetin, reserpine and tween-80 were purchased from Sigma-Aldrich.

### Preparation of HSP-NPs

The fabrication method utilized in this study is a modified version of the approach originally developed by Kakran et al. [21]. In this improved process, hesperidin is initially ground into a fine powder using a mortar to increase the surface area and facilitate dissolution. The finely ground hesperidin is subsequently dissolved in 99% ethanol at a concentration of 5 mg per 1 ml in a flask. Next, a buffer solution with a pH of 6.8 is prepared and added to the flask at a volume ratio of 2 ml per 100 mg of Hesperidin to maintain optimal pH conditions during synthesis. Tween 80, employed as a stabilizer to prevent particle aggregation, is added at a concentration of 1 µl per 1 ml of ethanol. To induce nanoparticle formation, deionized water is gradually introduced as an anti-solvent at a ratio of 35 ml per 1 ml of ethanol. This addition is performed slowly under continuous stirring using a magnetic stirrer for 30 min to ensure uniform mixing and particle nucleation. Following the anti-solvent precipitation, the resultant suspension is subjected to cooling centrifugation at 5 °C and 15,000 rpm for 20 min to effectively separate the nanoparticles from the supernatant. The cooling step helps maintain particle stability and prevents aggregation during centrifugation. The modifications introduced in this fabrication process enhance nanoparticle synthesis by promoting better control over particle size and stability, making the approach more efficient and reproducible compared to the original method.

### Preparation of QUR-NPs

The fabrication method employed in this study for the synthesis of quercetin nanoparticles is based on a modified anti-solvent precipitation approach, similar to techniques reported by Kakran et al. [22] and other subsequent studies. The process begins with dissolving quercetin powder (100 mg) in ethanol (99%) at a concentration of 5 mg per 1 ml. This step is performed in a clean, dry beaker under continuous stirring at 600 rpm for 30 min using a magnetic stirrer to ensure complete dissolution of the quercetin. The ethanol serves as a good solvent for quercetin due to its ability to dissolve hydrophobic compounds efficiently. To enhance the stability of the nanoparticles and prevent aggregation, Tween 80 is added as a stabilizer at a concentration of 1 µl per 1 ml of ethanol. The addition of Tween 80 ensures that the particles remain well dispersed and maintains colloidal stability throughout the process. In some cases, the mixture is subjected to ultrasonication for 5 min to further break down any potential aggregates and achieve better molecular dispersion. Following the dissolution and stabilization of quercetin, an anti-solvent precipitation technique is employed to induce nanoparticle formation. Deionized water (700 ml per 20 ml of ethanol) is slowly introduced to the ethanol solution as an anti-solvent under constant stirring at 1000 rpm. The slow and gradual addition of water causes the ethanol solubility of quercetin to decrease, leading to the formation of fine nanoparticles through controlled nucleation and particle growth. A buffer solution with a pH of 6.8 is also added at a volume ratio of 2 ml per 100 mg of quercetin to maintain optimal pH conditions during precipitation. The suspension obtained from the anti-solvent precipitation process is subjected to cooling centrifugation at 5 °C and 15,000 rpm for 20 min to separate the quercetin nanoparticles from the supernatant. Cooling is essential during centrifugation to minimize aggregation and maintain the physical stability of the nanoparticles. After centrifugation, the supernatant is discarded, and the nanoparticle pellet is washed with cold deionized water to remove residual ethanol and stabilizer. The washing step is typically repeated twice to ensure the purity of the nanoparticles. The resulting nanoparticle suspension is then dried using lyophilization (freeze-drying) or spray drying to obtain a fine, dry powder of quercetin nanoparticles. The dried nanoparticles are stored in airtight containers at 4 °C to maintain their stability and prevent moisture uptake. This modified fabrication process enhances the solubility, stability, and bioavailability of quercetin nanoparticles, making them suitable for pharmaceutical and biomedical applications. This method not only improves the yield and stability of quercetin nanoparticles but also offers simplicity and scalability, making it an effective strategy for producing bioactive nanoparticle formulations [22].

### TEM Images of HSP-NPs and QUR-NPs

To examine the size and morphology of the nanoparticles, transmission electron microscopy (TEM) was utilized. A drop of the particle suspension, which had been diluted and sonicated, was subjected to negative staining with 1% (w/v) uranyl acetate for 10 min. Subsequently, the stained sample was carefully deposited onto a carbon-coated copper TEM grid and left to air dry. Imaging was performed using a JEOL-2100 transmission electron microscope operating at an accelerating voltage of 100 kV.

### Experimental Animals

Thirty-two male Wistar albino rats were used in the present study. Their weights ranged from 200 to 250 g. Animals were purchased from National Research Centre animal house, Egypt. The animals were left for 5 days before carrying out the experiments to accommodate with the lab conditions which were 12/12 h light/dark cycle and 25 ºC temperature with free access to food and water. Animal’s care and experimental design were approved by the Proposal Research Ethics Committee, Faculty of Science Ain Shams University and the approval number was ASU-SCI/PHYS/2023/3/1.

### Experimental Design

Animals were divided into four groups. Control rats (*n* = 8) received daily i.p. injections of isotonic saline solution (0.9%) for 25 days. Starting from the 26th day, rats received isotonic saline solution two times (i.p. followed by oral administration) for another 21 days. Rat model of depression (*n* = 8) that was induced by daily i.p. of reserpine (0.2 mg/kg) for 25 days. On the 26^th,^ rats were administered i.p. with reserpine (0.1 mg/kg) and oral isotonic saline solution for another 21 days [23]. Rat model of depression treated with HNPs (*n* = 8) that received daily i.p. of reserpine (0.2 mg/kg) for 21 days. On the 26th day, rats received daily reserpine (0.1 mg/kg) followed by oral administration of HSNPS (50 mg/kg) [24] for another 21 days. The rat model of depression treated with QUR-NPs (*n* = 8) that received daily i.p. of reserpine (0.2 mg/kg) for 25. On the 26th day, rats received i.p. of reserpine (0.1 mg/kg) followed by oral administration of QNPs (50 mg/kg) [25] for another 21 days.

The following diagram showed the timeline of the experiment:

**Figure:**
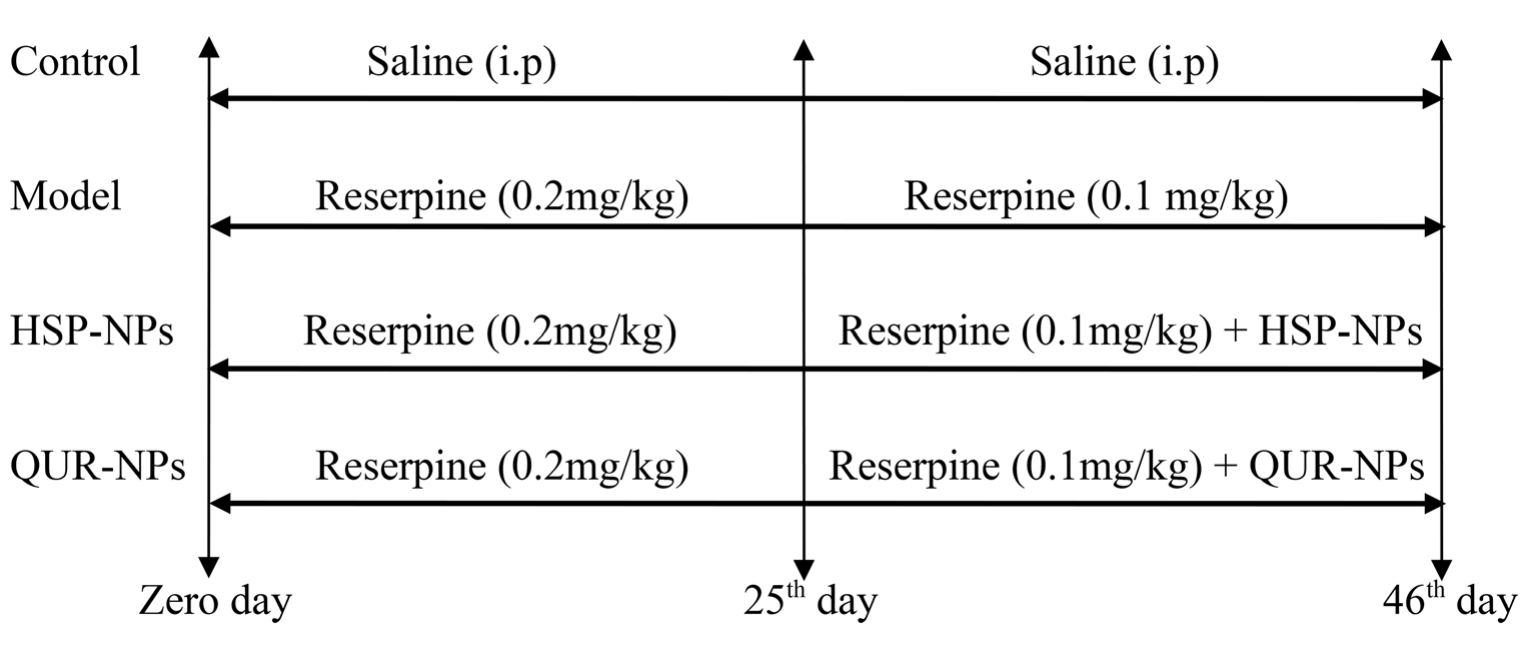

At the end of the experiment, which extended for 46 days, rats were sacrificed by sudden decapitation. The brain of each rat was removed and divided longitudinally into right and left halves on a cooled Petri dish. Then, each the cerebral cortex and hippocampus were dissected from each half. Each brain region was weighed and kept frozen at -80 ºC until carrying out the neurochemical measurements. The right halves of the cortex and hippocampus were used to measure the monoamine neurotransmitters, and the left halves were homogenized in tris-Hcl buffer (pH, 7.4) and centrifuged at 4000 rpm and 4° C for 15 min. then, the supernatant of each brain region was used to measure the oxidative stress parameters, MAO, AchE, Na, K,ATPase, and BDNF.

### Oxidative Stress Parameters

#### Measurement of Lipid Peroxidation

Lipid peroxidation was assayed by evaluating the malondialdehyde (MDA), one of the most important products of lipid peroxidation. This method is based on the interaction between the Thiobarbituric acid and the produced MDA producing pink color on boiling. The absorbance of this color was read at 532 nm [26].

#### Measurement of Nitric Oxide

Nitric oxide (NO) assay depends on measuring nitrite, which is produced by oxidizing the unstable gas NO. In this method, nitrite interacts with the Griess reagent, forming a purple color. Its absorbance is read at 450 nm [27].

#### Reduced Glutathione

Reduced glutathione (GSH) assay depends on reducing the Elman reagent by the sulfodryl (-SH) group of GSH. This produces 2-nitro-s-mercaptobenzoic acid with a light-yellow color that was read at 412 nm [28].

### Determination of Enzymes Activities

#### Na, K,ATPase

The activity of Na, K,ATPase was determined by subtracting the activity of Mg, ATPase from the total ATPase to obtain the Na, K,ATPase activity according to the method of Tsakiris et al. [29].

#### Acetylcholinesterase

The enzymatic activity of acetylcholinesterase (AchE) was assayed using the method described by Gorun et al. [30] This method depends on the hydrolysis of acetylthiocholine iodide by AChE forming thiocholine. The latter product reduces the sulfhydryl group (–SH) in DTNB reagent producing yellow colored thionitrobenzoic acid which is read at 412 nm.

#### Monoaminoxidase

Monoamine oxidase (MAO) activity was assayed depending on the hydrolysis of benzylamine by MAO into benzaldehyde, whose absorbance was measured at 280 nm [31].

### Determination of Brain-Derived Neurotrophic Factor (BDNF)

BDNF was determined in the cortex and hippocampus using EIZA kit purchased from DLdevelop (Wuxi Donglin Sci &Tech Development Co., Ltd). The microtiter plate provided in this kit has been pre-coated with an antibody specific to BDNF. Standards or samples are then added to the appropriate microtiter plate wells with a biotin-conjugated antibody preparation specific to BDNF. Next, Avidin conjugated to Horseradish Peroxidase (HRP) is added to each microplate well and incubated. After TMB substrate solution is added, only those wells that contain BDNF, biotin-conjugated antibody and enzyme-conjugated Avidin will exhibit a change in color. The enzyme-substrate reaction is terminated by the addition of sulphuric acid solution and the color change is measured spectrophotometrically at a wavelength of 450 nm ± 10 nm. The concentration of BDNF in the samples is then determined by comparing the optical density (O.D.) of the samples to the standard curve.

### Determination of Monoamines

Each of the cerebral cortex and hippocampus was homogenized in ice-cooled acidified alcohol. Then, the homogenate was centrifuged at 2000 rpm for 5 min. The supernatant was added to heptane and 0.2 N acetic acid. The mixture was vortexed and centrifuged at 2000 rpm for 5 min. the supernatant was used to evaluate the level of serotonin (5-HT), norepinephrine (NE) and dopamine (DA) using the spectrofluorometer (Jasco FP- 6500, JASCO Ltd., Tokyo, Japan) supplied with a xenon arc lamp source 150 W having an excitation slit bandwidth of excitation monochromator and emission slit bandwidth of emission monochromator of 5 nm each [32].

### Statistical Analyses

The data obtained in the present study were analyzed statistically by one-way analysis of variance (ANOVA) using SPSS software. That data was expressed as mean ± SEM. The difference between groups was considered significant when p-value < 0.05. Then Dunkan post-hoc test was performed to compare between groups.

## Results

Transmission electron microscopy (TEM) images of HSP-NPs and QUR-NPs.

**Fig. 1:**
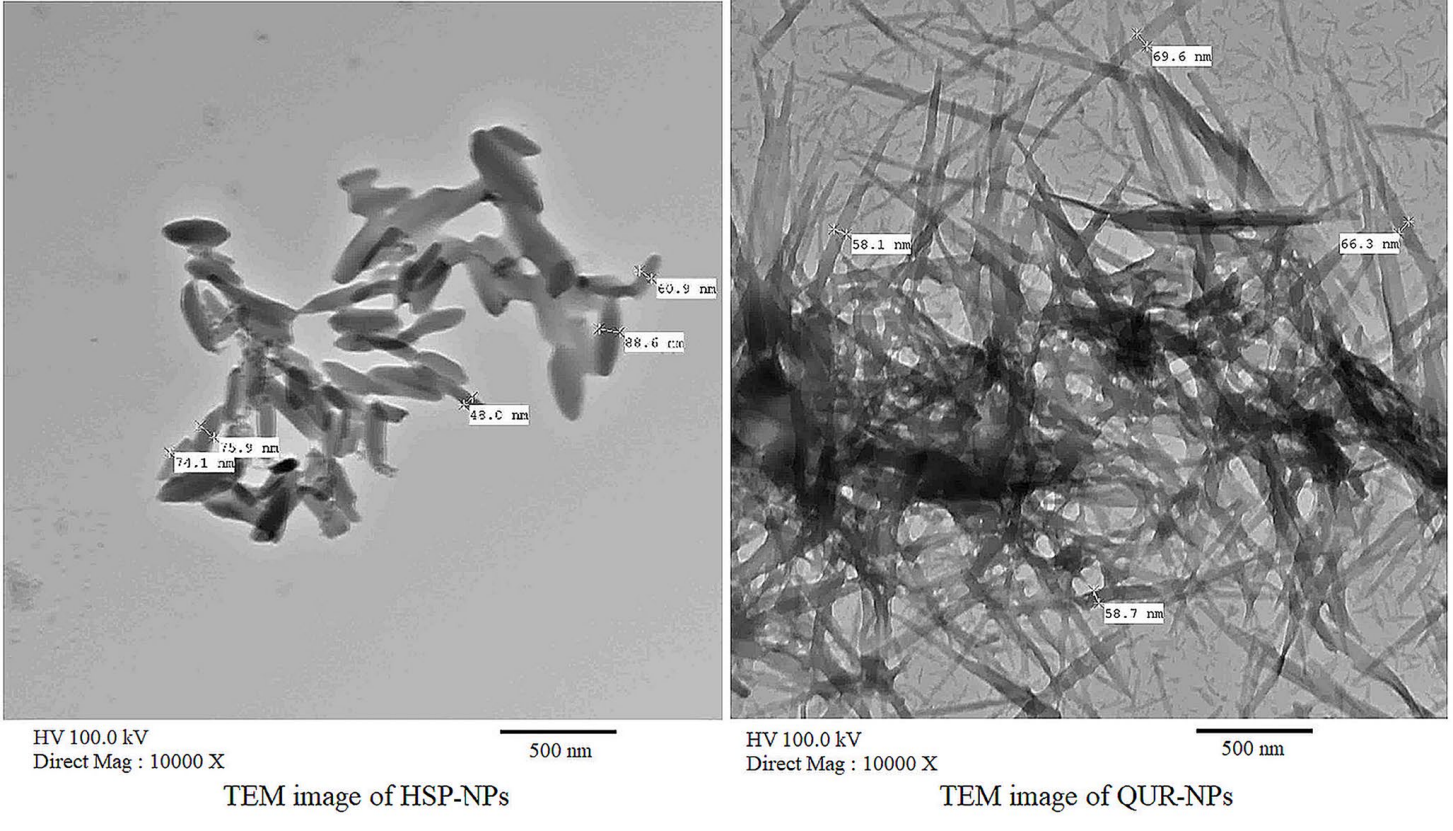
TEM image of HSP-NPs and QUR-NPs

TEM images showed that the average particle size of HSP-NPs was 75.7 nm and QUR-NPs was 63.2 (Fig. 1).

### Effects of HSP-NPs and QUR-NPs Treatment on Oxidative Stress Markers

As shown in Table 1, in the rat model of depression induced by reserpine, the level of malondialdehyde (MDA) in cortical tissues has been significantly increased by 23.3% compared to the control group. In contrast, in the group of rats with reserpine-induced depression and treated with HSP-NPs, the MDA level was not significantly different from the control group, with a non-significant increase of only 0.72%. Similarly, in the group of rats with reserpine-induced depression and treated with quercetin nanoparticles, the MDA level was significantly decreased compared to the untreated depression model group. However, it remained significantly elevated by 10.3% compared to the control group. The same findings were obtained in the hippocampal tissues, where MDA significantly increased by 30.1% in the hippocampus of the rat model of depression group on animals, however, both HSP-NPs and QUR-NPs were able to recover the MDA level to control-like values with a non-significant decrease of 13.9% and 3.7%, respectively. These results indicate that treatment with HSP-NPs nanoparticles was able to completely normalize the elevated MDA levels in the depression model, restoring them to near-control values in both the cortex and hippocampus brain regions of diseased rats. The quercetin nanoparticle treatment also demonstrated a marked reduction in oxidative stress, as evidenced by the significant decrease in MDA compared to the untreated depression group, but did not fully return MDA to control levels in cortical tissues.

**Table 1:** The effect of daily treatment with hesperidin nanoparticles (HES-NPs) and Quercetin nanoparticles (QUR-NPs) on the levels of malondialdehyde (MDA) (nmol/g), nitric oxide (NO) ((µmol/g), and reduced glutathione (GSH) (mmol/g) in the cortex and the hippocampus of rat model of depression induced by reserpine

		Control	Depressed rats	%D	HES-NPs	%D	QUR-NPs	%D	*p*-value
Cortex	MDA	4.16^a^ ± 0.11	5.13^b^ ± 0.13	+ 23.32	4.19^a^ ± 0.11	+ 0.72	4.59^c^ ± 0.14	+ 10.34	0.000
	NO	0.19^a^ ± 0.017	0.12^b^ ± 0.015	-36.84	0.18^a^ ± 0.014	-5.26	0.20^a^ ± 0.023	+ 5.26	0.018
	GSH	5.26^a^ ± 0.23	4.17^b^ ± 0.06	-20.72	5.12^a^ ± 0.09	-2.66	5.57^a^ ± 0.20	+ 5.89	0.000
Hippocampus	MDA	5.12^a^ ± 0.35	6.66^b^ ± 0.59	+ 30.08	4.41^a^ ± 0.29	-13.87	4.93^a^ ± 0.33	-3.71	0.003
	NO	0.41^a^ ± 0.04	0.27^b^ ± 0.04	-34.15	0.30^ab^ ± 0.02	-26.83	0.42^a^ ± 0.05	+ 2.44	0.028
	GSH	12.06^a^ ± 0.38	9.47^b^ ± 0.29	-21.48	11.05^a^ ± 0.67	-8.37	11.89^a^ ± 0.69	-1.41	0.007

Values represent the mean ± S.E.M

Statistically significant means (p-value $\:\text{<}$ 0.05) are given different letters

%D is the percentage difference = [(Group value - Control value)/Control value] ×100

In the rat model of depression induced by reserpine, a significant reduction was observed in the levels of both nitric oxide (NO) and glutathione (GSH) in the cortex and hippocampus. Specifically, NO levels were attenuated by 36.8% in the cortex and 34.2% in the hippocampus, while GSH levels were decreased by 20.7% in the cortex and 21.5% in the hippocampus compared to control levels. Treatment with either HSP-NPs and QUR-NPs for 2 weeks was able to recover the levels of NO and GSH in both brain regions to non-significant control-like levels. However, the restoration was more effective with HSP-NPs treatment than with QUR-NPs in the hippocampus. In the hippocampus, HSP-NPs treatment did not fully return the NO level to the complete control level, although it was significantly improved compared to the untreated depression model. These findings indicate that the depression-induced model was associated with significant reductions in both NO and antioxidant GSH in the cortex and hippocampus. The administration of HSP-NPs and QUR-NPs was effective in normalizing these alterations, with HSP-NPs showing a slightly more potent effect, particularly in the hippocampus region (Table 1).

### Effects of HSP-NPs and QUR-NPs Treatment on Enzyme Activities

**Fig. 2:**
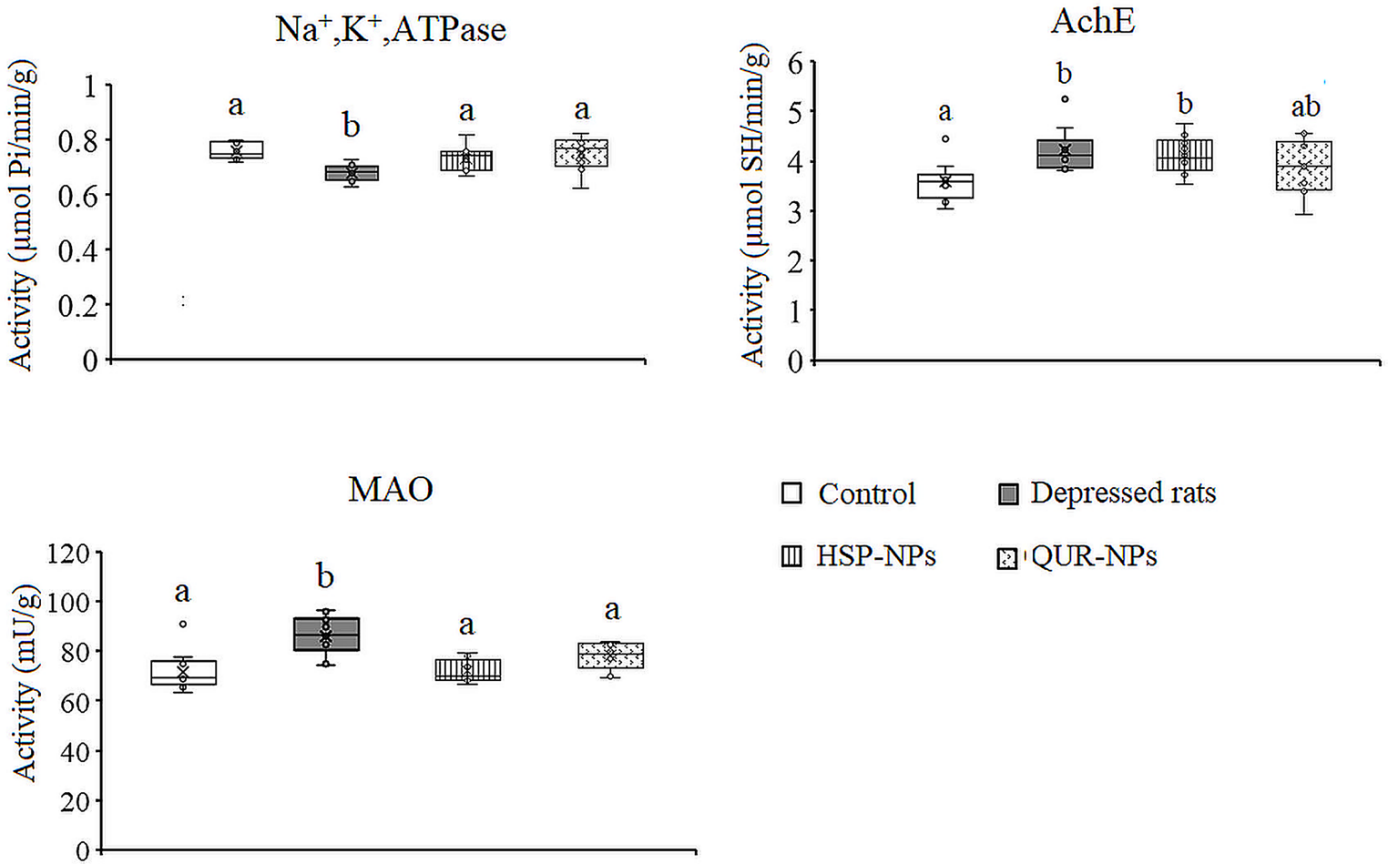
Effect of HSP-NPs and QUR-NPs on the activity of Na, K,ATPase, acetylcholinesterase (AchE) and monoamine oxidase (MAO) in the cortex of rat model of depression induced by reserpine. Statistically significant means (p-value $\:\text{<}$ 0.05) are given different letters and statistically significant means are given similar letters

**Fig. 3:**
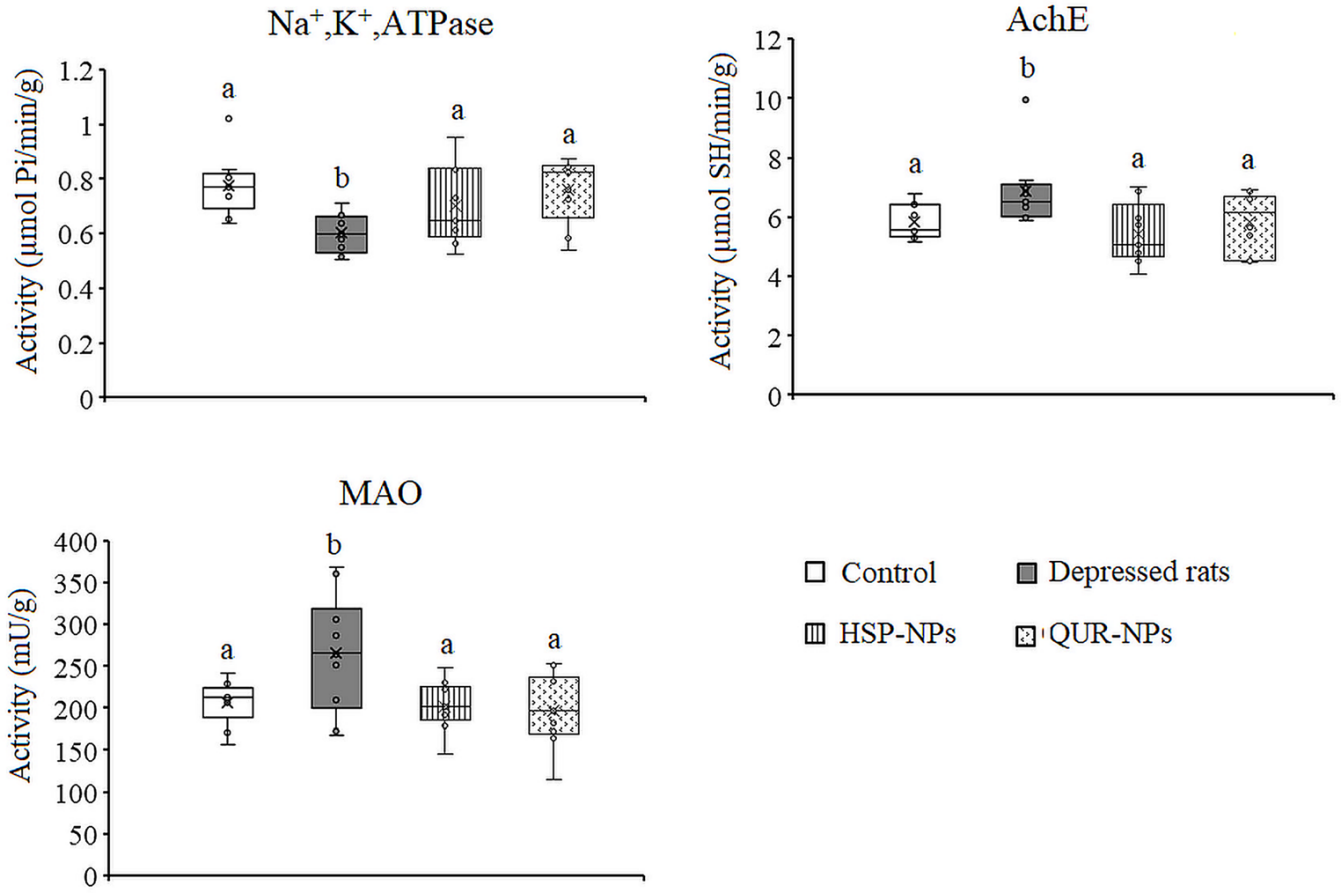
Effect of HSP-NPs and QUR-NPs on the activity of Na, K,ATPase, acetylcholinesterase (AchE) and monoamine oxidase (MAO) in the hippocampus of rat model of depression induced by reserpine. Statistically significant means (p-value $\:\text{<}$ 0.05) are given different letters and statistically significant means are given similar letters

In Figs. 2 and 3, ANOVA showed that the activity of the Na^+^,K^+^-ATPase enzyme was significantly decreased in the cortex (*p* = 0.003) and hippocampus (*p* = 0.014) of the rat model of depression compared to the control group. Specifically, the cortical activity of Na^+^,K^+^-ATPase was attenuated by 14.5%, while in the hippocampus, the activity was reduced by 23.1% in the depression model animals compared to control values. This reduction in Na^+^,K^+^-ATPase activity can have detrimental effects on neuronal function and ionic homeostasis, potentially contributing to the pathophysiology of depression. Treatment with either HSP-NPs and QUR-NPs recovered the enzyme activity in both the cortex and hippocampus to control-like levels.

In the rat model of depression induced by reserpine, a significant increase was observed in the activity of both AchE (*p* = 0.016, *p* = 0.033) and MAO (*p* = 0.001, *p* = 0.007) in the cortex and hippocampus respectively. Specifically, AchE activity was elevated by 17.5% in the cortex and 17.4% in the hippocampus, while MAO activity was elevated by 20% in the cortex and 28.7% in the hippocampus, compared to control levels. Daily treatment with HSP-NPs or QUR-NPs for two weeks, restored the cortical and hippocampal MAO activity and he hippocampal AchE. In the cortex, HSP-NPs failed to restore AchE activity while QUR-NPs improved the change in AchE activity induced by reserpine (Figs. 2 and 3).

### Effects of HSP-NPs and QUR-NPs Treatment on the Trophic Factor

**Fig. 4:**
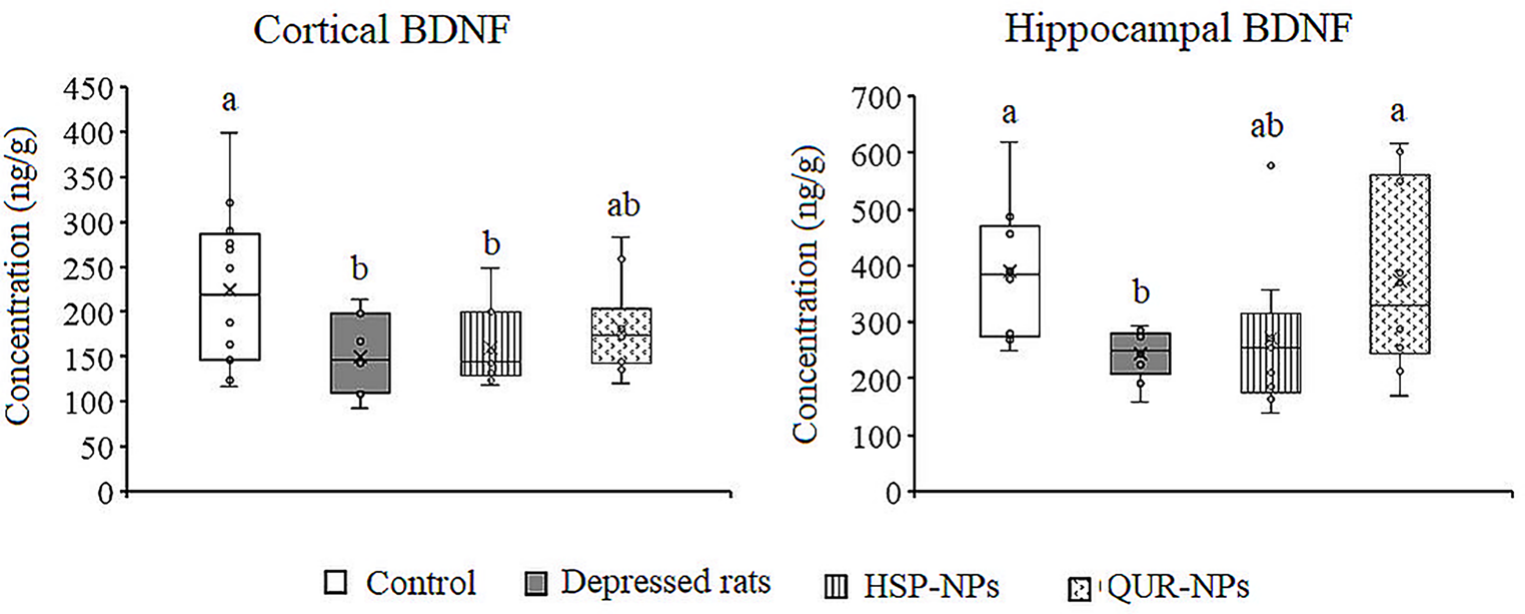
Effect of HSP-NPs and QUR-NPs on the level of brain derived neurotrophic factor (BDNF) in the cortex and hippocampus of rat model of depression induced by reserpine. Statistically significant means (p-value $\:\text{<}$ 0.05) are given different letters and statistically significant means are given similar letters

ANOVA revealed a significant decrease in BDNF levels in the cortex (*p* = 0.034) and hippocampus (*p* = 0.036) of the reserpine-induced depression model. Compared to controls, BDNF levels were attenuated by 33.2% and 37.3% in the cortex and hippocampus, respectively. HSP-NPs and QUR-NPs treatment improved BDNF levels in both brain regions. However, only quercetin nanoparticles fully restored hippocampal BDNF levels to control values (Fig. 4).

### Effects of HSP-NPs and QUR-NPs Treatment on Monoamines

**Fig. 5:**
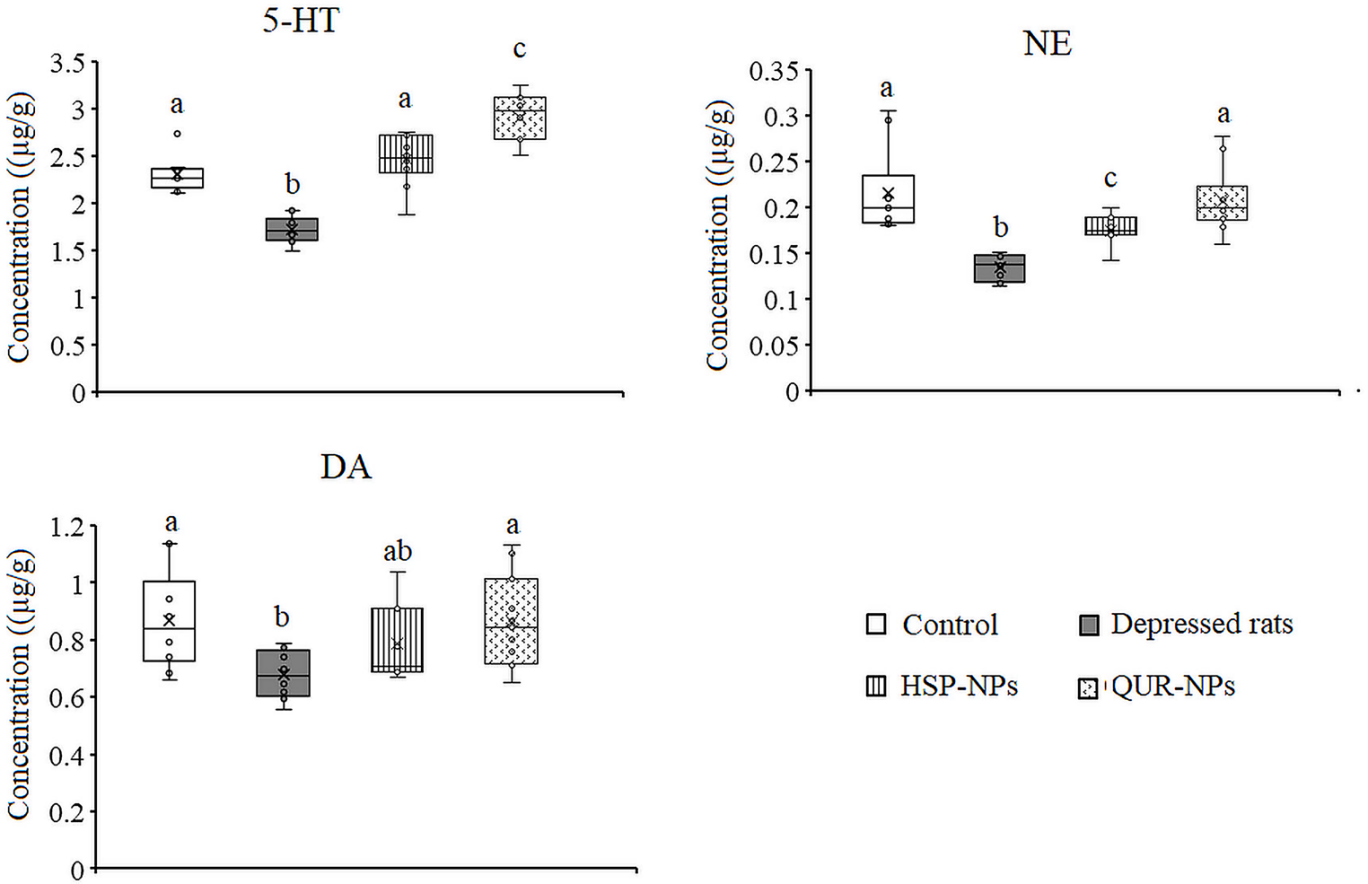
Effect of HSP-NPs and QUR-NPs on the levels of serotonin (5-HT), norepinephrine (NE) and dopamine (DA) in the cortex of rat model of depression induced by reserpine. Statistically significant means (p-value $\:\text{<}$ 0.05) are given different letters and statistically significant means are given similar letters

**Fig. 6:**
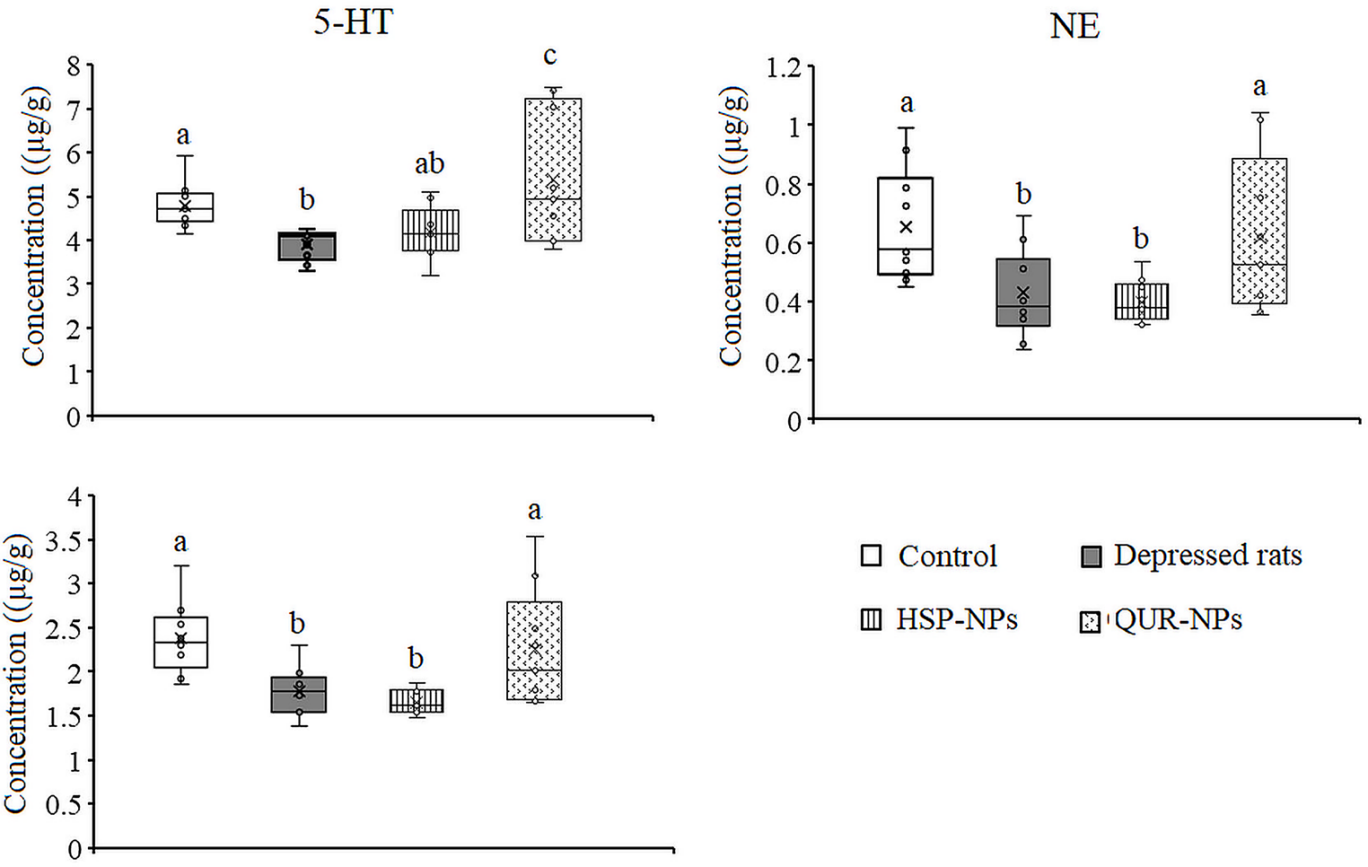
Effect of HSP-NPs and QUR-NPs on the levels of serotonin (5-HT), norepinephrine (NE) and dopamine (DA) in the hippocampus of rat model of depression induced by reserpine. Statistically significant means (p-value $\:\text{<}$ 0.05) are given different letters and statistically significant means are given similar letters

Figures 5 and 6 summarize the effects of HSP-NPs and QUR-NPs treatment on monoamine (5-HT, NE, and DA) levels in the cortex and hippocampus of a depression model. Reserpine, a monoamine-depleting agent, significantly reduced 5-HT (*p* = 0.001, *p* = 0.005), NE (*p* = 0.001, *p* = 0.006), and DA (*p* = 0.031, *p* = 0.002) levels in both brain regions compared to the control group. Daily treatment with HSP-NPs for two weeks restored the cortical 5-HT and improved the levels of NE and DA. In the hippocampus, HSP-NPs improved 5-HT while the decreased levels of NE and DA induced by reserpine were unchanged. In the cortex and hippocampus, daily treatment with QUR-NPs for two weeks potentially reversed the decreased 5-HT level to a significant increase as compared to the control and the rat model of depression and restored the levels of NE and DA to control like value.

## Discussion

Quercetin and hesperidin as flavonoids exert neuroprotective effects on the brain by influencing neuropathological factors such as oxidative stress, and inflammation [33]. Furthermore, they showed the ability to modulate some neuro-enzymatic activities and neurotransmitter levels in different brain regions. However, the limited bioavailability of these active components hinders its therapeutic efficacy when administered in their native form. Therefore, nanoparticles of hesperidin and quercetin have been developed, in the present study, to enhance their bioavailability and therapeutic potential.

Nanoparticle formulations of these native compounds offer several advantages in terms of dosage, effectiveness, and side effects compared to traditional administration of native materials [34]. The enhanced bioavailability of nanoparticles allows for lower doses to be administered while maintaining therapeutic efficacy. This, in turn, reduces the risk of toxicity and side effects associated with high drug doses. The controlled release of nanoparticles may also improve their effectiveness and provide a sustained drug level in the body [35].

In the present study, reserpine administration resulted in elevated oxidative stress markers in the cortical and hippocampal tissues of the animal model. This increase in oxidative stress can be attributed to multiple mechanisms associated with reserpine’s effects on monoamines and mitochondrial function. One primary mechanism involves the inhibition of the vesicular monoamine transporter (VMAT2) by reserpine [23]. VMAT2 is responsible for packaging monoamine neurotransmitters, such as dopamine, norepinephrine, and serotonin, into vesicles for release [36]. Thus, VMAT2 inhibition leads to an upregulation of monoamine metabolism and their breakdown by monoamine oxidase (MAO). As a result, reactive oxygen species (ROS) are produced during the metabolism of monoamines [37], contributing to oxidative stress. Additionally, reserpine-induced mitochondrial dysfunction may play a significant role in oxidative stress [38]. Mitochondria are crucial for energy production within cells but can also generate ROS as a byproduct. Reserpine disrupts the electron transport chain in mitochondria, impairing ATP synthesis and increasing ROS production [39]. The produced free radicals, especially singlet oxygen, can combine with NO, producing the powerful damaging molecule peroxynitrite [40] that can cause nitrolysation for protein and DNA and neuronal death [41]. This may explain the reduced level of NO in the two brain regions. Additionally, the present decrease in GSH, which is the main free radical scavenger in the brain, may be due to its exhaustion in neutralizing the ROS produced by reserpine.

Several reports suggest that oxidative stress is a major player in the pathogenesis of depression [11, 42]. The excessive generation of ROS and the lack of efficient antioxidant response can trigger processes such as neurodegeneration and neuronal death which could be related to the onset and progression of depression symptoms. By targeting oxidative stress, restoring monoamine balance, and modulating neuroenzyme activities, it may be possible to develop new strategies for treating depression and improving the overall well-being of individuals affected by this debilitating disorder.

In the present study, the administration of HSP-NPs and QUR-NPs on a daily basis for two weeks restored the levels of oxidative stress markers in the cortex and hippocampus. This beneficial effect can be attributed to their free radical scavenging activity [43, 44], which helps reduce lipid peroxidation. As a result, the levels of malondialdehyde (MDA), a byproduct of lipid peroxidation, are attenuated. Accordingly, PSP-NPs and QUR-NPs keep the integrity of cell membranes and mitigate oxidative stress-induced damage in the brain.

Moreover, HSP-NPs and QUR-NPs have been shown to enhance the synthesis and availability of glutathione (GSH) [45, 46], which plays a crucial role in neutralizing free radicals and maintaining cellular redox balance. By increasing GSH levels, these flavonoids enhance the cellular antioxidant potential and support the defense system against oxidative stress induced by reserpine. Furthermore, the ability of HSP-NPs and QUR-NPs to limit the production of ROS prevented the formation of peroxynitrite. This was indicated from the control-like level of NO induced by the two agents.

In the present work, three enzyme activities have been changed due to the administration of reserpine. There was upregulation in the MAO and AchE activity and attenuation in the Na^+^/K^+^-ATPase in the cortex and hippocampus of the rat model of depression.

The upregulated MAO activity can be attributed to the inhibitory effect of reserpine on VMAT2. This effect inhibits the reuptake of 5-HT, NE, and DA and increases their enzymatic degradation in the cytosol. This explains the recorded cortical and hippocampal depletion of monoamines, which is the main cause of depression. Additionally, the increased AChE activity agrees with the study of Tiwari et al. [47] and Ullas Kamath et al. [48], who observed increased AChE activity in depression patients. This might be a compensatory mechanism employed by the body to counteract the excess of acetylcholine in the body. Elevated ACh levels in the CNS may contribute to depressive symptoms [49]. One theory regarding depressive disorders development is the catecholaminergic–cholinergic balance theory. An increase in ACh activity may cause a decrease in dopamine activity and thus an increase in depressive symptoms [50, 51].

On the other hand, treatment of reserpine model of depression with HPS-NPs and QUR-NPs restored the cortical and hippocampal changes in the enzymatic activities to control-like values except for AChE activity, which remained affected after QUR-NPs treatment in the cortical tissue. This effective treatment ability of both HSP-NPs and QUR-NPs could be attributed to their neuroprotective effects, including the preservation of neuronal structure and function, and to their antioxidant effects, which help protect enzymes from oxidative dysregulation. However, the incomplete restoration of AchE may be related to the incomplete recovery in the monoamine’s neurotransmitters and the imbalance between the acetylcholine and catecholamines.

It has been reported that the reduction of Na+, K+-ATPase activity seems to be an important characteristic of depressive disorders [17, 52]. The present decrease in Na, K,ATPase could be correlated to the decreased 5-HT, NE, and DA, which have a regulatory role on the pump activity [53, 54] and to the oxidative stress. Thus, the depletion of neurotransmitters caused by reserpine can disrupt this regulatory mechanism, potentially altering Na^+^/K^+^-ATPase. Therefore, the ability of HSP-NPs and QUR-NPs to restore the cortical and hippocampal Na^+^/K^+^-ATPase could be attributed to the improved monoamine levels and to their antioxidant effects.

Decreased BDNF levels in the cortex and hippocampus of reserpine rat model of depression was observed. The depletion of monoamine neurotransmitters caused by reserpine can lead to reduced BDNF expression and secretion [55]. Furthermore, this action may also interfere with BDNF signaling pathways. BDNF acts through its receptor, TrkB, to activate downstream signaling cascades involved in neuronal survival and plasticity [56]. Reduced monoamine levels caused by reserpine can impair BDNF-TrkB signaling [57], leading to downstream effects on neuronal function and plasticity. Moreover, changes in BDNF mRNA levels in response to reserpine treatment have been reported. These alterations in BDNF gene expression may contribute to the observed changes in BDNF protein levels in the present study.

Our findings highlight the challenge of restoring BDNF levels to control-like values, particularly in the cortex in animals treated with HSP-NPs and QUR-NPs. However, intriguingly, quercetin nanoparticles demonstrated the ability to restore BDNF to control-like value in the hippocampus tissue of treated animals. It is well-established that oxidative stress is associated with decreased BDNF levels [11, 58]. Therefore, the partial improvement observed in BDNF levels obtained in the tissues of treated animals could be attributed to the antioxidant capacity and the neuroprotection offered by HSP-NPs and QUR-NPs. These properties likely contributed to the reduction of oxidative stress and protection of neurons from damage, ultimately enhancing their survival.

The mitigating effects of HSP-NPs and QUR-NPs on depressive-associated dysregulation in neuronal parameters are evident in the levels of monoamine neurotransmitters in both cortex and hippocampus tissue. HSP-NPs and QUR-NPs restored some of the neurotransmitters to their control-like values. It is worth noting that, QUR-NPs exhibited more pronounced effects on neurotransmitters compared to HSP-NPs in both brain areas. The exact mechanism of restoring the monoamine neurotransmitters by antioxidant nanoparticles is not yet fully understood. However, it is known that hesperidin and quercetin have an inhibitory action on MAO [6, 59], which could mediate the restoration of neurotransmitter levels. Furthermore, the role played by these nanoparticles in modulating the activity of neurotransmitter transporters may contribute to the reuptake of neurotransmitters from the synaptic cleft, thereby increasing their levels in neurons. Additionally, the modulation of enzymes involved in neurotransmitter synthesis and the regulation of some neurotropic factors by hesperidin or quercetin may also play a crucial role in the restoration of monoamine neurotransmitters.

## Conclusion

In conclusion, HSP-NPs and QUR-NPs have potential in mitigating oxidative stress, restoring enzyme activity, and improving BDNF and neurotransmitter levels in the cortex and hippocampus of rats treated with reserpine. Hesperidin and quercetin, antioxidants, can scavenge free radicals, protecting neurons from damage and preserving their functionality. HPS-NPs and QUR-NPs inhibit MAO enzymes, enhancing the availability of monoamine neurotransmitters, serotonin, norepinephrine, and dopamine. They also influence Na, K-ATPase, and AChE enzymes, restoring neurotransmitter balance and synaptic communication. The differential effect of HSP-NPs and QUR-NPs may be attributed to factors like structure-activity relationship, bioavailability, and interaction with target molecules. Accordingly, it could be suggested that both HSP-NPs and QUR-NPs could offer more robust restorative action against molecular dysregulation associated with depression. However, it is important to note that while preclinical studies have shown promising results, further research is necessary to fully elucidate the underlying molecular mechanisms and confirm the efficacy and safety of HSP-NPs and QUR-NPs in mitigating depressive-like symptoms induced by reserpine. Clinical trials and rigorous investigations are needed to translate these findings into potential therapeutic interventions for depression and related disorders in humans.

## Data Availability

No datasets were generated or analysed during the current study.
